# Ni (II), Cu (II) and Zn  (II) Metal Chelates With Some Thiazole Derived Schiff-Bases: Their Synthesis, Characterization and Bactericidal Properties

**DOI:** 10.1155/MBD.1999.75

**Published:** 1999

**Authors:** Zahid H. Chohan

**Affiliations:** Department of Chemistry Islamia University Bahawalpur Pakistan

## Abstract

A number of thiazole derived tridentate Schiff-bases (LH) and its metal chelates of the type [M(L)_2_X] where M=Ni(II), Cu(II) and Zn(II), L=substituted salicylaldehyde (5-H, 5-CH_3_, 5-OCH_3_, 5-NO_2_ and 5-Cl) and X=CI have been synthesized and characterized with the help of elemental analyses, conductivity measurements, magnetic moments, UV-Vis, IR and NMR spectral data. An octahedral structure for Ni(II) and Zn(II) and a distorted octahedral structure for Cu(II) chelates have been proposed. All the Schiff-bases and their metal chelates have been screened for their biological activity against *Escherichia coli*, *Staphylococcus aureous* , *Pseudomonas aeruginosa* and *Klebsiella pneumonae* and in comparison, the metal chelates have been shown to possess more antibacterial activity than the uncomplexed Schiff-bases.


### Ni(II), Cu(II) AND Zn(II) METAL CHELATES WITH SOME THIAZOLE DERIVED SCHIFF-BASES: THEIR SYNTHESIS, CHARACTERIZATION AND BACTERICIDAL PROPERTIES

Zahid H. Chohan

Department of Chemistry, Islamia University, Bahawalpur, Pakistan

**ABSTRACT**

A number of thiazole derived tridentate Schiff-bases (LH) and its metal chelates of the type $[M(L_2)X]$ where M=Ni(II), Cu(II) and Zn(II), L=substituted salicylaldehyde (5-H, 5-$CH_3$, 5-$OCH_3$, 5-$NO_2$ and 5-Cl) and X=Cl have been synthesized and characterized with the help of elemental analyses, conductivity measurements, magnetic moments, UV-Vis, IR and NMR spectral data. An octahedral structure for Ni(II) and Zn(II) and a distorted octahedral structure for Cu(II) chelates have been proposed. All the Schiff-bases and their metal chelates have been screened for their biological activity against *Escherichia coli*, *Staphylococcus aureous, Pseudomonas aeruginosa* and *Klebsiella pneumonae* and in comparison, the metal chelates have been shown to possess more antibacterial activity than the uncomplexed Schiff-bases.

### INTRODUCTION

Schiff-bases and the relevant transition metal complexes are still found to be of great interest in co-ordination chemistry[1-4] although this subject has been extensively studied[5-8]. A number of reviews have appeared on the co-ordination chemistry of Schiff-base complexes[9-11] which exhibit an interesting variety of stereochemical behaviors in this area. Thiazole and substituted thiazoles possess interesting biological activity probably conferred to them by the strong aromaticity of their ring system[12], which leads to great *in vivo* stability. When diverse functional groups that interact with biological receptors are attached to this ring, compounds possessing outstanding properties could be obtained[13]. With a view to understanding such potentialities and also, the usefulness of Schiff-bases the present author has undertaken the syntheses of some thiazole derived Schiff-bases (Fig 1) by condensing some substituted salicylaldehydes (5-H, 5-$CH_3$, 5-$OCH_3$, 6-$NO_2$ and 5-Cl) with 2-aminothiazole and their metal chelates (1-15) with Ni(II), Cu(II) and Zn(II). In order to establish the biological role of metals, these prepared Schiff-bases and their metal chelates have been screened for their bactericidal activity against bacterial species such as *Escherichia coli, Staphylococcus aureous, Pseudomonas aeruginosa* and *Klebsiella pneumonae*.

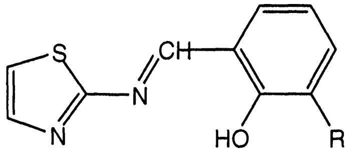

R = H, $CH_3$, $OCH_3$, $NO_2$ or Cl

### EXPERIMENTAL

### Material and methods

All chemicals and solvents used were of Analar grade. All the metals were used as their metal(II) chlorides. 2-Aminothiazole and substituted salicylaldehyde were obtained from Aldrich. IR, $^1H$ NMR and $^{13}C$ NMR spectra were recorded on a Philips Analytical PU 9800 FTIR and Brucker 250 MHz instruments. UV-Visible spectra were obtained on a Hitachi U-2000 double-beam spectrophotometer. Conductance of the metal complexes was determined in DMF at $10^{-3}$ dilution on a YSI-32 model conductometer. Magnetic measurements were done on solid complexes using the Gouy method. The synthesized Schiff-bases and their metal chelates were analyzed for C, H and N by microanalytical techniques and the metal contents in the chelates were estimated by standard methods. Melting points were recorded on a Gallenkamp apparatus and are uncorrected. The antibacterial studies were carried out with the help of the Department of Pathology, Quaid-e-Azam Medical College, Bahawalpur, Pakistan.

### Preparation of the Schiff-bases

An amount of salicylaldehyde (0.01 M) was dissolved in hot ethanol (20 mL). To this solution was added ethanolic solution of 2-aminothiazole (0.01 M, 30 mL). Then 2-3 drops of conc $H_2SO_4$ were added in it and the mixture, refluxed with stirring for 1 h and then left for 24 h at room temperature. During this period yellow needles precipitated.The crystals were filtered, washed with ethanol and dried at 60°C

**Preparation of the Metal Complexes**

An ethanol solution of appropriate metal(II) chloride (1 mmol, 20 mL) was added to a stirred hot ethanol solution of the respective Schiff-base (2 mmol, 30 mL). The resulting mixture was refluxed for 3 h. The solution was then cooled, filtered, reduced to nearly half its volume and then left for two days at room temperature. During this period the product crystallized. It was filtered, washed with ethanol and ether and dried to give desired metal complexes (**1-15**).

**Antibacterial Studies**

**Preparation of Discs.**

The Schiff base/complex (30 µg) in DMF (0.01mL) was applied on a paper disc, [prepared from blotting paper (3 mm diameter)] with the help of a micropipette. The discs were left in an incubator for 48 h at 37° C and then applied on the bacteria grown agar plates.

**Preparation of Agar Plates.**

Minimal agar was used for the growth of specific bacterial species. For the preparation of agar plates for *Escherichia coli*, MacConkey agar (50 g), obtained from Merck, was suspended in freshly distilled water (1 L). It was allowed to soak for 15 minutes and then boiled on a water bath until the agar was completely dissolved. The mixture was autoclaved for 15 minutes at 120° C and then poured into previously washed and sterilized Petri dishes and stored at 40° C for inoculation.

**Procedure of Inoculation.**

Inoculation was done with the help of a platinum wire loop which was made red hot in a flame, cooled and then used for the application of bacterial strains.

**Application of Discs.**

A sterilized forceps was used for the application of paper disc on the already inoculated agar plates. When the discs were applied, they were incubated at 37° C for 24 h. The diameter of the zone of inhibition was then measured.

Table 1 Physical, Spectral and Analytical Data of the Schiff bases

| Schiff base/ Mol. Form. | M.P. (°C) | IR ($cm^{-1}$) | Calc (Found %) C H N |
|---|---|---|---|
| **R=H** $C_{10}H_8N_2OS$ [204.18] | 163 | 2900(0H),1635 (HC=N),1285(C-O) | 58.8 3.9 13.7 (58.5) (4.2) (14.1) |
| **R=$CH_3$** $C_{11}H_{10}N_2OS$ [218.19] | 178 | 2905(0H),1630 (HC=N),1285(C-O) | 60.5 4.6 12.8 (60.6) (4.9) (12.7) |
| **R=$OCH_3$** $C_{11}H_{10}N_2O_2S$ [234.19] | 155 | 2900(0H),1635 (HC=N),1280(C-O) | 56.4 4.3 12.0 (56.3) (4.5) (11.8) |
| **R=$NO_2$** $C_{10}H_{10}N_3O_3S$ [212.18] | 186 | 2900(0H),1630 (HC=N),1280(C-O) | 56.6 4.7 19.8 (56.6) (4.6) (20.1) |
| **R=Cl** $C_{10}H_7ClN_2OS$ [238.63] | 172 | 2905(0H),1635 (HC=N),1285(C-O) | 50.3 2.9 11.7 (50.2) (2.6) (11.5) |

### RESULTS AND DISCUSSION

**Physical properties**

The Schiff-bases were prepared by condensation of equimolar amounts of substituted salicylaldehyde and aminothiazole in ethanol. The crystallized products after characterization (Table 1, 2) were used further for the preparation of their metal complexes.

All the complexes (**1-15**) prepared are crystalline solids and melt with decomposition above 250° C without showing sharp melting points. The complexes are soluble in chloroform, DMF, DMSO and benzene. Their melting behaviour, solubility and crystalline nature suggests that they are non-polymeric. Elemental analyses data (Table 3) suggested 1:2 (metal:ligand) stoichiometry. Low conductance values (12-17 $ohm^{-1}$ $cm^2$ $mol^{-1}$) indicated that the complexes are non-electrolytic[14,15] in nature.

Table 2 $^{1}H$ NMR and $^{13}C$ NMR Data of the Schiff bases

| R | $^{1}H$ NMR (DMSO-$d_6$) (ppm) | $^{13}C$ NMR (DMSO-$d_6$) (ppm) |
|---|---|---|
| H | 6.9(m,2H,aromatic),7.3(m,1H,aromatic), 7.5(m,1H,aromatic),7.9(s,1H,HC=N),7.4 (d,1H,heteroaromatic),7.8(d,1H,hetero-aromatic),12.8(s,1H,OH). | 116.5,118.2,118.7(aromatic),131.6, 132.3,160.7(aromatic),118.6,143.2, 155.9(heteroaromatic),165.2(HC=N). |
| $CH_3$ | 2.3(s,3H,$CH_3$).7.1(m,2H,aromatic),7.3(m, 1H,aromatic),7.5(m,1H,aromatic),8.0(s,1 H,HC=N),7.4(d,1H,heteroaromatic),7.8(d, 1H,heteroaromatic),12.9(s,1H,OH). | 31.1($CH_3$),116.6,118.2,118.5(aromatic) 131.6,132.3,160.7(aromatic),118.6, 143.2,155.9(heteroaromatic),165.2 (HC=N). |
| $OCH_3$ | 3.8(s,3H,$CH_3$),6.9(m,2H,aromatic),7.3(m, 1H,aromatic),7.5(m,1H,aromatic),8.0(s,1 H,HC=N),7.4(d,1H,heteroaromatic),7.8(d, 1H,heteroaromatic),12.8(s,1H,OH). | 31.1($OCH_3$),116.6,118.2,118.5(aromatic), 131.6,132.3,160.7(aromatic),118.6, 143.2,154.9(heteroaromatic),165.2 (HC=N). |
| $NO_2$ | 6.9(m,2H,aromatic),7.4(m,1H,aromatic), 7.5(m,1H,aromatic),8.1(s,1H,HC=N),7.4 (d,1H,heteroaromatic),7.8(d,1H,hetero-aromatic),13.0(s,1H,OH). | 116.6,118.2,118.5(aromatic),131.6,132. 3,165.7(aromatic),118.6,143.2,155.9 (heteroaromatic),165.2(HC=N). |
| Cl | 6.8(m,2H,aromatic),7.4(m,1H,aromatic), 7.5(m,1H,aromatic),8.1(s,1H,HC=N),7.4 (d,1H,heteroaromatic),7.8(d,1H,hetero-aromatic),12.9(s,1H,OH). | 31.1(CH3),116.6,118.2,118.5(aromatic)1 31.6,132.3,160.7(aromatic),118.6,143.3, 155.9(heteroaromatic),165.2(HC=N). |

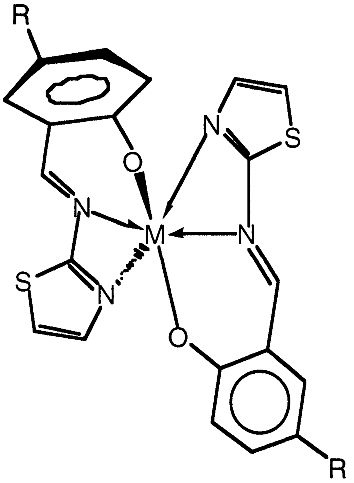

R = H, $CH_3$, $OCH_3$, $NO_2$, Cl

M = Ni(II), Cu(II), Zn(II)

Figure 2: Structure proposed for the metal chelates

**Infrared and NMR spectra**

The IR spectra of the ligands (Table 1) show a weak and broad band at ~ 2900 $cm^{-1}$ instead of a strong band at 3100 $cm^{-1}$ (expected due to phenolic –OH group).This might be due to intramolecular hydrogen bonding between the hydroxyl hydrogen and nitrogen of the azomethine group forming a six membered ring[16]. The absence of νOH in the complexes suggested the deprotonation of the phenolic –OH of the Schiff base and its co-ordination through the O atom. Moreover, the νC-O and νC=N modes which occured at ~ 1285 and ~ 1640 $cm^{-1}$ respectively in the Schifff bases were shifted in the complexes. This shifting of νC-O towards higher frequency (~ 1330-1342 $cm^{-1}$) and lowering of νC=N (~ 1580-1610 $cm^{-1}$) suggested that the co-ordination of the Schiff-bases occurred through the deprotonated oxygen of the phenolic –OH group and nitrogen of the azomethine (HC=N) groups[16]. Further conclusive evidence of the co-ordination of the ligands with the metals was confirmed by the appearance of weak low frequency bands at ~ 480-510 and ~ 345-370

$cm^{-1}$ (Table 4) due to metal-oxygen and metal-nitrogen stretching vibrations[17-21] in the metal complexes and not observable in the spectra of Schiff-bases.
The NMR spectra are also proved to have considerable diagnostic values when compared with the reported[22] related values. According to $^{1}H$ and $^{13}C$ NMR spectra features of the Schiff-bases (Fig 1) are shown in Table 2 which are in agreement with the proposed structures.

Table 3 Physical Data of Metal Chelates

| No | Metal chelate/ Mol. Formula | M.P(°C) (dec.) | B.M. ($\mu_{eff}$) | Calc (Found)% C H N |
|---|---|---|---|---|
| 1 | $[Ni(L^1)_2(Cl)_2]$ $C_{20}H_{14}NiCl_2N_4O_2S_2$ [535.93] | 252-254 | 3.07 | 44.8 2.6 10.4 (44.5) (2.5) (10.5) |
| 2 | $[Ni(L^2)_2(Cl)_2]$ $C_{22}H_{18}NiCl_2N_4O_2S_2$ [563.95] | 248-250 | 3.12 | 46.9 3.2 9.9 (47.0) (3.6) (10.2) |
| 3 | $[Ni(L^3)_2(Cl)_2]$ $C_{22}H_{18}NiCl_2N_4O_4S_2$ [595.95] | 273-275 | 3.10 | 44.33 3.0 9.4 (44.67) (2.9) (9.3) |
| 4 | $[Ni(L^4)_2(Cl)_2]$ $C_{20}H_{12}NiCl_2N_6O_6S_2$ [625.93] | 266-268 | 3.13 | 38.37 1.9 13.4 (38.71) (2.0) (13.6) |
| 5 | $[Ni(L^5)_2(Cl)_2]$ $C_{20}H_{12}NiCl_3N_4O_2S_2$ [569.38] | 257-260 | 3.13 | 42.5 2.1 9.8 (42.3) (2.2) (9.2) |
| 6 | $[Cu(L^1)_2(Cl)_2]$ $C_{20}H_{14}CuCl_2N_4O_2S_2$ [540.96] | 255-257 | 1.83 | 44.4 2.6 10.4 (44.8) (2.3) (10.1) |
| 7 | $[Cu(L^2)_2(Cl)_2]$ $C_{22}H_{18}CuCl_2N_4O_2S_2$ [568.78] | 251-253 | 1.90 | 46.45 3.2 9.8 (46.18) (3.9) (9.7) |
| 8 | $[Cu(L^3)_2(Cl)_2]$ $C_{22}H_{18}CuCl_2N_4O_4S_2$ [600.78 | 271-273 | 1.88 | 44.0 3.0 9.3 (44.7) (3.5) (9.2) |
| 9 | $[Cu(L^4)_2(Cl)_2]$ $C_{20}H_{12}CuCl_2N_6O_6S_2$ [630.76] | 268-270 | 1.95 | 38.0 2.1 13.3 (38.1) (2.5) (13.1) |
| 10 | $[Cu(L^5)_2(Cl)_2]$ $C_{20}H_{12}CuCl_3N_4O_2S_2$ [574.21] | 252-254 | 1.96 | 41.8 2.1 9.8 (41.9) (2.0) (9.5) |
| 11 | $[Zn(L^1)_2(Cl)_2]$ $C_{20}H_{14}ZnCl_2N_4O_2S_2$ [542.59] | 274-276 | Dia | 44.3 2.6 10.3 (44.4) (2.5) (10.9) |
| 12 | $[Zn(L^2)_2(Cl)_2]$ $C_{22}H_{18}ZnCl_2N_4O_2S_2$ [570.61] | 262-264 | Dia | 46.3 3.2 9.8 (46.5) (3.1) (9.2) |
| 13 | $[Zn(L^3)_2(Cl)_2]$ $C_{22}H_{18}ZnCl_2N_4O_4S_2$ [602.61] | 257-259 | Dia | 43.8 3.0 9.3 (43.4) (3.6) (9.2) |
| 14 | $[Zn(L^4)_2(Cl)_2]$ $C_{20}H_{12}ZnCl_2N_6O_6S_2$ [632.59] | 269-171 | Dia | 38.0 1.9 13.3 (38.2) (2.2) (13.0) |
| 15 | $[Zn(L^5)_2(Cl)_2]$ $C_{20}H_{12}ZnCl_3N_4O_2S_2$ [576.04] | 278-280 | Dia | 41.7 2.1 9.7 (41.9) (2.5) (10.1) |

**UV-Visible spectra and Magnetic moments**

Electronic spectra of the Ni(II) complexes exhibit four bands at 8000-9885, 13545-15555, 23775-24100 and 27245-29300 $cm^{-1}$. The first three bands were assigned to thespin allowed transitions $^{3}A_{2}g$ ($\nu_1$) $\rightarrow$ $^{3}T_{2}g$ , $^{3}A_{2}g$ ($\nu_2$) $\rightarrow$ $^{3}T_{1}g$ (F) and $^{3}A_{2}g$ ($\nu_3$)$\rightarrow$ $^{3}T_{1}g$ (P) respectively. The fourth band at 27245-29300 $cm^{-1}$ was of high intensity and was due to ligand-metal charge-transfer. The occurence of three spin-allowed transitions supports the octahedral geometry for the Ni(II) complexes[23]. The magnetic moments of the complexes lie in the range 3.06-3.13 B.M (Table 4) and alsoconfirms the octahedral geometry[24].

The electronic spectra of the Cu(II) complexes exhibit two bands, a broad unsymmetrical band in the visible region at 14530-15100 $cm^{-1}$ may be due to $^2E_g \rightarrow ^2T_{2g}$ transitions in the octahedral geometry and a sharp band of high intensity at 26500-28170 $cm^{-1}$ assigned to Logan-metal charge-transfer[24]. The Cu(II) complexes of tetrahedral geometry or octahedral geometry[25,26] normally exhibit magnetic moments in the range ~ 1.9 B.M. The magnetic moments of the present Cu(II) complexes lie in the range 1.81-1.96 B.M. On the basis of electronic spectra and magnetic susceptibility data, a distorted octahedral geometry[27] is proposed for the complexes. The electronic spectra of the Zn(II) complexes exhibit only a high intensity band at 27250-28500 $cm^{-1}$ assigned[28] to ligand-metal charge-transfer.

Table 4 Spectral and Analytical Data of Metal Chelates

| No | IR ($cm^{-1}$) | $\lambda_{max}$ ($cm^{-1}$) |
|---|---|---|
| 1 | 1330(C-O),1610(HC=N),480(M-O),345(M-N) | 29205,23775,13545,8245 |
| 2 | 1335(C-O),1580(HC=N),485(M-O),355(M-N) | 28255,24100,15555, |
| 3 | 1342(C-O),1585(HC=N),495(M-O),350(M-N) | 29300,23890,14285 |
| 4 | 1340(C-O),1605(HC=N),498(M-O),365(M-N) | 28375,24005,14972 |
| 5 | 1341(C-O),1602(HC=N),510(M-O),345(M-N) | 27650,23995,15345 |
| 6 | 1337(C-O),1590(HC=N),512(M-O),370(M-N) | 27245,14530 |
| 7 | 1335(C-O),1595(HC=N),505(M-O),367(M-N) | 26500,14877 |
| 8 | 1340(C-O),1610(HC=N),492(M-O),365(M-N) | 28170,15100 |
| 9 | 1342(C-O),1605(HC=N),495(M-O),355(M-N) | 27270,14655 |
| 10 | 1338(C-O),1595(HC=N),480(M-O),345(M-N) | 27220,14995 |
| 11 | 1335(C-O),1610(HC=N),495(M-O),370(M-N) | 27250 |
| 12 | 1342(C-O),1600(HC=N),510(M-O),365(M-N) | 28445 |
| 13 | 1335(C-O),1610(HC=N),510(M-O),360(M-N) | 28500 |
| 14 | 1337(C-O),1598(HC=N),505(M-O),361(M-N) | 27780 |
| 15 | 1340(C-O),1595(HC=N),500(M-O),365(M-N) | 27555 |

Table 5 Antibacterial Activity Data

| Schiff-base/-Chelate | Microbial Species | | | |
|---|---|---|---|---|
| | a | b | c | d |
| $HL^1$ | ++ | + | + | ++ |
| $HL^2$ | + | ++ | ++ | + |
| $HL^3$ | ++ | + | - | + |
| HL4 | + | ++ | + | + |
| HL5 | ++ | + | + | ++ |
| 1 | +++ | +++ | +++ | +++ |
| 2 | +++ | +++ | +++ | +++ |
| 3 | +++ | ++++ | +++ | ++ |
| 4 | +++ | ++ | ++ | ++ |
| 5 | ++++ | +++ | +++ | +++ |
| 6 | +++ | +++ | +++ | ++ |
| 7 | ++++ | +++ | +++ | +++ |
| 8 | ++ | +++ | + | ++ |
| 9 | +++ | +++ | ++++ | +++ |
| 10 | +++ | +++ | +++ | +++ |
| 11 | +++ | ++++ | ++ | +++ |
| 12 | ++ | ++++ | +++ | +++ |
| 13 | +++ | +++ | +++ | ++ |
| 14 | ++++ | +++ | ++ | ++++ |
| 15 | +++ | ++ | ++ | +++ |

a=*Escherichia coli*, b= *Staphylococcus aureus*,
c= *Pseudomonas aeruginosa* d=*Klebsiella pneumonae*

Inhibition zone diameter mm (% inhibition): +, 6-10 (27-45 %); ++, 10-14 (45-64 %); +++, 14-18 (64-82 %); ++++, 18-22 (82-100 %). Percent inhibition values are relative to inhibition zone (22 mm) of the most active compound with 100 % inhibition.

In the light of the above discussion octahedral structures for Ni(II) and Zn(II) complexes and distorted octahedral structure for Cu(II) complexes is proposed. It is tentatively proposed that the Schiff-base ligands coordinate through the nitrogen of the azomethine group, nitrogen of thiazole ring and oxygen of phenolic group forming a stable chelate ring structure (Fig 2).

**Antibacterial activity**

The antibacterial activity of Schiff-bases and their complexes was studied against *Escherichia coli*, *Staphylococcus aureous, Pseudomonas aeruginosa* and *Klebsiella pneumonae* bacterial species. Paper disc diffusion method devised and reported[29,30] earlier was adopted for screening. All the Schiff-bases and its complexes individually exhibited varying degrees of inhibitory effects on the growth of the tested bacterial species. The antibacterial results are reproduced in Table 5 which evidently show that the activity of the Schiff-bases became more pronounced when co-ordinated with the metals.

### References

1. A. Hills, D. L. Hughes, G. J. Leigh and J. R. Sanders, *J.Chem.Soc.Dalton Trans.*, 325 (1991).
2 R. Knoch, A. Wilk, K. J. Wannowius, D. Reinen and H. Elias, *Inorg.Chem.*, **29**, 3799 (1990).
3 L. Carbonaro, A. Giacometti, L. Senatore and L. Valli, *Inorg.Chim.Acta.*, **165**, 197 (1989).
4 F. A. Bottino, P. Finocchiaro and E. Libbertini., *J.Coord.Chem.*, **16**, 341 (1988).
5 R. H. Holm and G. W. Everett,Jr, *Progr.Inorg.Chem.*, **7**, 83 (1967).
6 Z.H.Chohan, S.K.A.Sherazi and M.Praveen, *Synth.React.Inorg.Met-Org.Chem.*, **28**, 1673 (1998).
7 Z.H.Chohan and H.Pervez, *Synth.React.Inorg.Met-Org.Chem.*, **23**, 1061 (1993).
8 Z.H.Chohan and A.Rauf, *Synth.React.Inorg.Met-Org.Chem.*, **26**, 591 (1996).
9 M. D. Hobday and T. D. Smith, *Coord.Chem.Rev.*, **7**, 83 (1967).
10 L. F. Lindoy, *Quart.Rev.*, **25**, 379 (1971).
11 M. C. Golonka and A. Bartecki, *Coord.Chem.Rev.*, **31**, 251 (1979).
12 G. Kornis, "Comprehensive Heterocyclic Chemistry", A. R. Katritzky Ed., Pregamon Press Vol. 6, Part 4B, 1984.
13 C. T. Supuran, M. Barboiu, C. Luca, E. Pop, M. E. Brewster and A. Dinculescu, *Eur.J.Med Chem.*, **31**, 596 (1996).
14 A.M.Shallary, M.M.Moustafa and M.M.Bekheit, *J.Inorg.Nucl.Chem.*, **41**, 267 (1979).
15 W.J.Geary, *Coord.Chem.Rev.*, 7, 81 (1971).
16 J. E. Kovacic, *Spectrochim Acta.*, **23A**, 183 (1967).
17 K. Nakamoto, "Infrared Spectra of Inorganic and Coordination Compounds", John Wiley, New York, 1963.
18 D. M. Adams, "Metal Ligand and Related Vibrations", Edward Arnold, London, 1967
19 R. A. Condrate and K. Nakamoto, *J.Chem.Phys.*, **42**, 2590 (1965).
20 J. F. Jackowitz, J. A. Durkin and J. G. Walter, *Spectrochim.Acta.*, **23A**, 67 (1967).
21 J. F. Jackowitz and J. G. Walter, *Spectrochim.Acta.*, **22**, 1393 (1966).
22 D.H.Williams and I.Fleming,"Spectroscopic Methods in Organic Chemistry", 4th Ed, Mc Graw Hill, London (1989).
23 A.B.P.Lever,"Inorganic Electronic Spectroscopy", 2nd Ed, Elsevier, N York, 1984.
24 R. S. Drago, "Physical Methods in Inorganic Chemistry", Reinhold, N. York, 1965.
25 B. N. Figgis, "Introduction to Ligand Fields", Interscience, N. York, 1966.
26 L. Sacconi, M. Ciampolini and V, Campigili, *Inorg.Chim.*, **4**, 407 (1965).
27 D.W.Meek, R.S.Drago and T.S.Piper, *Inorg.Chem.*, **1**, 285 (1962).
28 C.J.Balhausen,"Introduction to Ligand Field",Mc Graw Hill, New York, 1962.
29 Z.H.Chohan and A.Rauf, *J.Inorg.Biochem.*, **46**, 41 (1992).
30 Z.H.Chohan and S.Kausar, *Chem.Pharm.Bull.*, **40**, 2555 (1992).

**Received: January 28, 1999 - Accepted: February 3, 1999 - Received in revised camera-ready format: February 10, 1999**

